# Hereditary angioedema due to C1 inhibitor deficiency in Belarus: epidemiology, access to diagnosis and seven novel mutations in SERPING1 gene

**DOI:** 10.1186/s12948-021-00141-0

**Published:** 2021-04-07

**Authors:** Irina Guryanova, Chiara Suffritti, Debora Parolin, Andrea Zanichelli, Nastassia Ishchanka, Ekaterina Polyakova, Mikhail Belevtsev, Francesca Perego, Marco Cicardi, Yulia Zharankova, Natalya Konoplya, Sonia Caccia, Antonio Gidaro

**Affiliations:** 1 Belarusian Research Center for Pediatric Oncology, Hematology and Immunology, Minsk, Belarus; 2 General Medicine Department, ASST-Fatebenefratelli-Sacco, Milan, Italy; 3 Department of Biomedical and Clinical Sciences Luigi Sacco, Università Degli Studi Di Milano, Milan, Italy; 4 ASST Grande Ospedale Metropolitano Niguarda, Milan, Italy; 5 Department of Subacute Therapy, IRCCS-Istituti Clinici Scientifici Maugeri, Milan, Italy

**Keywords:** Belarus, C1 inhibitor, Hereditary angioedema (HAE), Rare disease, SERPING1 variants

## Abstract

**Background:**

Hereditary angioedema due to C1 inhibitor deficiency (C1-INH-HAE) is a rare disease. Few states in developing countries have an adequate management of HAE, but none of them belongs to the former USSR area. This study analyses data from C1-INH-HAE patients from Belarus.

**Methods:**

Data about clinical characteristics, genetics, access to diagnosis and treatment were collected from 2010 by the Belarusian Research Center for Pediatric Oncology, Hematology and Immunology in Minsk. A questionnaire about attacks, prophylactic (LTP) and on-demand therapy (ODT) was administered to patients.

**Results:**

We identified 64 C1-INH-HAE patients belonging to 26 families, 27 (42.2%) of which were diagnosed in the last 3 years. The estimated minimal prevalence was 1:148,000. Median age at diagnosis was 29 years, with diagnostic delay of 19 years. Thirty-eight patients answered a questionnaire about therapy. Eleven patients did not use any treatment to resolve HAE attacks. Twenty-seven patients underwent ODT: 9 with appropriate treatments, and 18 with inappropriate treatments. Nine patients used LTP with attenuated androgens and 1 with tranexamic acid. Thirty-two patients answered a questionnaire about attacks and triggers: 368 angioedema attacks were reported, with an average of 10 attacks per year. We found 24 different *SERPING1* variants: 9 missenses, 6 in splice sites, 6 small deletions, 2 nonsense, 1 large deletion; 7 have not been previously described. De novo* variants* were found in 11 patients.

**Conclusions:**

C1-INH-HAE diagnosis and management in Belarus is improved as seen from the high number of new diagnosis in the last 3 years. Next steps will be to reduce the diagnostic delay and to promote the LTP and ODT.

## Background

Hereditary angioedema (HAE) due to C1 Inhibitor (C1-INH) deficiency (C1-INH-HAE) is a rare autosomal dominant disease caused by reduced C1-INH plasma levels (C1-INH-HAE type I) or by the presence of a dysfunctional C1-INH (C1-INH-HAE type II) [1]. C1-INH-HAE type I is estimated to occur in approximately 85% of cases, type II occurs in the remaining 15%. C4 is reduced in both C1-INH-HAE type I and II while C3 is normal [2]. The disease is caused by sequence variants in C1-INH gene (*SERPING1,* OMIM #606860) that is located on chromosome 11 (11.q12-q13.1) and comprises of eight exons and seven introns distributed over 17 kb, with introns containing 17 repetitive Alu sequences (1,5,6). More than 560 different pathogenic variants in *SERPING1* have been described to date, ranging from single nucleotide substitutions, small insertions and/or deletions, large deletions and duplications (HGMD® Professional, http://www.hgmd.cf.ac.uk/ac/index.php) [3]. De novo variants, i.e. variants where parents have got a normal C1-INH, can be identified in approximately 25% of the families [4]. The deficiency of C1-INH results in uncontrolled activation of the contact system and release of bradykinin, the mediator of increased vascular permeability and angioedema manifestations [5, 6]. C1-INH-HAE manifests with recurrent episodes of edema of the skin, gastrointestinal tract and upper airway. The disease is disabling and laryngeal edema can lead to asphyxiation and death if left untreated [2]. Data on the prevalence of C1-INH-HAE are sparse. Globally, the estimated frequency reported in literature varies from 1 every 10,000 to 1 every 50,000 people without racial or gender differences [7–9]. Focusing on European countries, the higher prevalence is reported in England [10], Denmark [11, 12], Italy [13], Hungary [14], Norway [15], Sweden [16], and Switzerland [17], where the calculated prevalence is 1:65,000 to 1:75,000 inhabitants. Lower rate is reported in Greece [14], Serbia [18], Slovenia [19], and Spain [20], where the prevalence has been evaluated to be approximately 1:90,000 to 1:105,000 inhabitants.

Since C1-INH-HAE is a genetic disease, the deficiency of C1-INH is present from birth. Nevertheless, a minority of patients have perinatal angioedema symptoms. Patients typically begin to present clinical manifestations in childhood and attacks frequency often increases during puberty. Before the second decade of life the majority of patients manifest symptoms of angioedema [21]. Due to the rarity of the disease and to the fact that the clinical symptoms overlap with those of other forms of angioedema, C1-INH-HAE is frequently misdiagnosed. Consequently, C1-INH-HAE patients may experience considerable delay between symptoms onset and diagnosis [21].

In a recent international observational study analysing data of patients eligible to Icatibant treatment (Icatibant Outcome Survey, IOS) conducted in 8 European countries, the mean delay in diagnosis of C1-INH-HAE patients was 12.8 years [22].

Currently on-demand therapies (ODT) for the treatment of C1-INH-HAE attacks are available: fresh frozen plasma, two plasma derived C1-INH (Berinert®, Cinryze®), one recombinant C1-INH (Ruconest®) and the bradykinin receptor antagonist Icatibant (Firazyr®) [23]. Clinical guidelines recommend patients to treat all attacks at onset [23]. ODT reduce the duration of attacks and the risk of death due to asphyxia. Patients who do not reach adequate disease control with ODT can be switched to long-term prophylaxis (LTP) [23], with attenuated androgens, subcutaneous preparation of plasma-derived C1-INH [24] or subcutaneous Lanadelumab (Takhzyro®, a human monoclonal antibody to kallikrein) [25].

Data regarding epidemiology, genetics, access to diagnosis and therapy from states belonging to former Republics of the Union of Soviet Socialist Republics are missing. This study analyses data from C1-INH-HAE patients from Belarus, a country born in 1991, as an example of the region.

## Materials and methods

### Patients

Patients diagnosed with C1-INH-HAE at the Belarusian Research Center for Pediatric Oncology Hematology and Immunology in Minsk since 2010 have been included in the study. For each patient, the following data were collected from the medical records: date of birth, age at symptoms onset, date of diagnosis, plasma levels of complement parameters. Diagnosis of C1-INH-HAE was based on personal and/or family history of angioedema and on C1-INH functional or antigenic plasma levels ≤ 50% of normal, as proposed in the guidelines for the diagnosis of C1-INH-HAE [2, 23, 26, 27]. Patients were diagnosed as C1-INH-HAE type I when functional and antigenic C1-INH were ≤ 50% of normal. Patients were classified as C1-INH-HAE type II when functional C1-INH was ≤ 50% and antigenic C1-INH was > 50% of normal. Independent families were identified, and the first diagnosed member of a family was considered as proband. Thirty-eight patients answered a questionnaire about prophylactic and on-demand therapy and thirty-two patients about attacks and triggers during the previous year from the date of participation in the study. All patients provided their consent to use their data anonymously. This study was conducted in accordance with the Declaration of Helsinki and was approved by the Ethics Committee of the Belarusian Research Center for Pediatric Oncology Hematology and Immunology in Minsk (w/n 20/11/19).

### Complement parameters

C1-INH, C3, C4, C1q were measured at diagnosis. Patients were not on prophylactic treatment when complement parameters were measured. C1-INH levels (normal range: 0.21–0.39 g/l) were measured using the Dade Behring BN ProSpec (Siemens, Germany); C4 (normal range: 0.1–0.4 g/l 1–11 years and 0.2–0.5 g/l > 11 years) and C3 (normal range: 0.6–1.5 g/l 0–3 years, 0.7–1.8 g/l 3–6 years, 0.9–1.8 g/l > 6 years), using the Konelab Primer 60i (Thermo Scientific, USA). The Infosheet Anthos multimode fluorometer Zenyth 1100 (Italy) was used to measure functional C1-INH (normal range: 77.3–128.8%) and C1q (normal range: 2–20 RU/ml) levels.

### *SERPING1* gene mutational screening

DNA was extracted from peripheral white blood cells using standard protocols. To identify Single Nucleotide Variants (SNV), exonic regions of *SERPING1* were amplified from samples of total genomic DNA. Primary mutational screening was performed by Single Strand Conformational Polymorphism (SSCP). DNA fragments with abnormal mobility compared to wild type control were subsequently analysed by Sanger sequencing. Regarding Copy Number Variation (CNV) analysis of *SERPING1*, we used Multiplex Ligation-dependent Probe Amplification (MLPA) technique. Capillary electrophoresis was performed on ABI 3130 Genetic Analyzer, (Applied Biosystem, USA).

We also used custom Next Generation Sequencing (NGS) Panel (Illumina, USA) of 347 genes most frequently mutated in patients with Primary Immune Deficiency (PID) and HAE. Sequencing was performed on MiSeq platform (Illumina, USA). Observed variants were confirmed by Sanger resequencing. Data interpretation included population filtering, functional prediction (SIFT, PolyPhen2, Hope) and other necessary steps according to Joint Recommendation of the Association for Molecular Pathology and the College of American Pathologists [28].

### Statistical analysis

Descriptive analysis was performed using median and percentage distribution of the population of enrolled patients, as appropriate. The data were analysed using the SPSS statistical package, version 24.00 (SPSS INC., Chicago, IL, US).

## Results

### Epidemiology and access to diagnosis

**Fig. 1:**
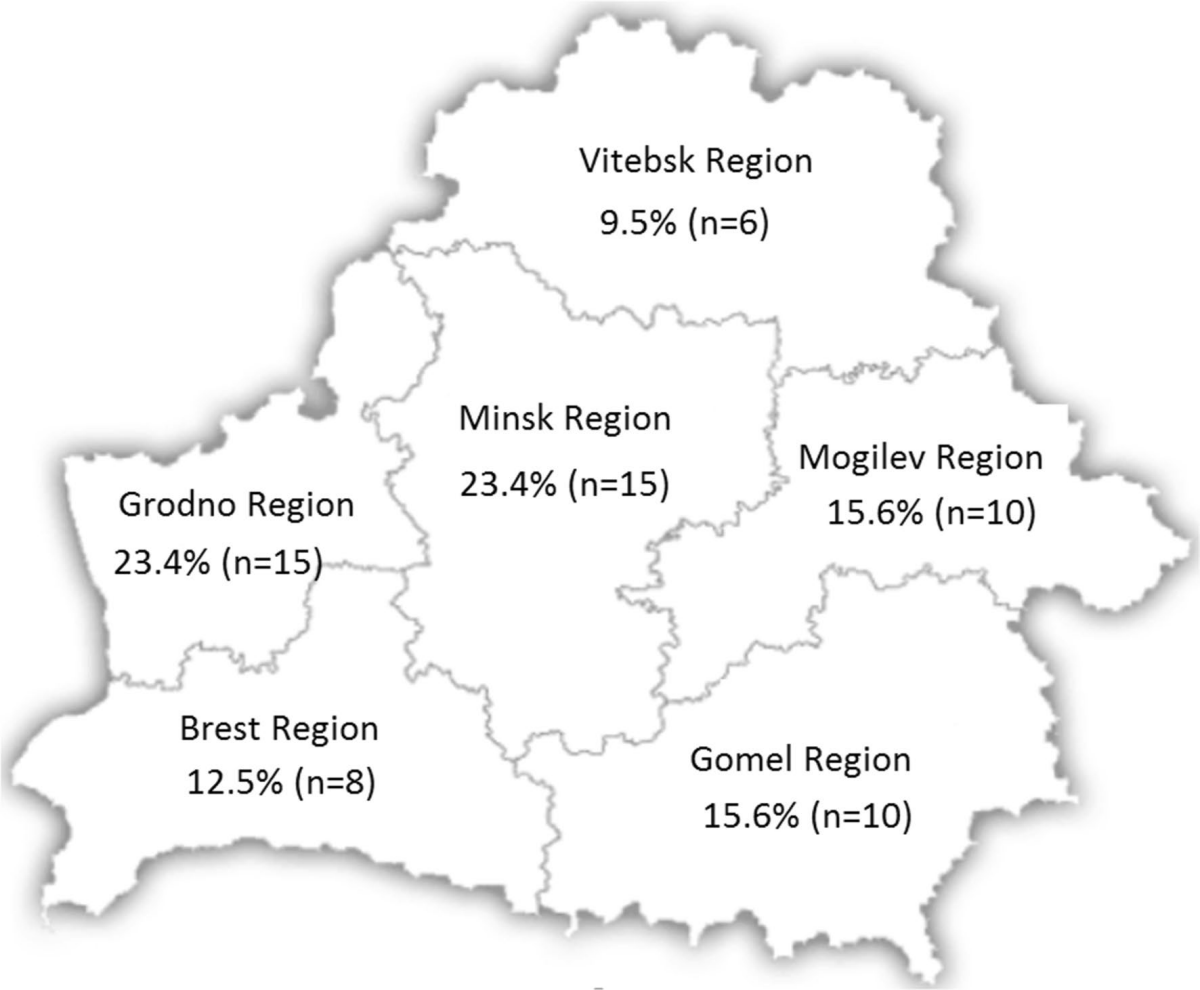
Geographical distribution of C1-INH-HAE patients in The Republic of Belarus. The percentage of individuals diagnosed for C1-INH-HAE is indicated for each Belarus region (the exact number is specified in parentheses)

We identified 64 C1-INH-HAE patients (39 female): 54 were type I (84.4%) and 10 type II (15.6%). Based on these results, the estimated minimal prevalence of HAE in Belarus is 1:148,000. In 27 (42.2%) patients (8 of them were type II C1-INH-HAE) diagnosis was performed in the last 3 years. Thus, poor clinical awareness can contribute to the low diagnostic rate of HAE and may be a confounder in ascertaining true national prevalence. Geographical location of the patients in the country is shown in Fig. 1.

Median age of patients was 31.5 (min 2–max 68 years), median age at diagnosis was 29.0 (min 1–max 65 years). Median of C4 antigen was 0.08 g/l and was ≤ 50% of normal in 92.2% of patients. Median of antigenic C1-INH was 0.06 g/l and was ≤ 50% of normal in 100% of type 1 patients. Functional C1-NH was ≤ 40% of normal in 100% of patients. Median of C3 antigen was 1.04 g/l and was normal or slightly lower than normal range in 98.4% of patients. Levels of C1q were normal in 100% of patients. Data about mean age of patients, symptom’s onset, diagnosis and diagnostic delay, laboratory assessments are summarized in Table 1. Eight patients had been diagnosed before the first symptoms appeared, based on *SERPING1* gene analysis.

**Table 1:** Epidemiology of the 64 C1-INH-HAE patients identified in Belarus

	C1-INH-HAE population	Type I C1-INH-HAE	Type II C1-INH-HAE
Mean age (SD)	33.2 (2.4)	32 (2.6)	39.8 (5.1)
Mean age of symptom’s onset (SD)	12.3 (1.2)	12.6 (1.4)	11.2 (1.5)
Mean age at diagnosis (SD)	30.7 (2.4)	29.2 (2.6)	38.6 (5.2)
Median of diagnostic delay (IQ_25_-IQ_75_)	19.3 (2.4)	12.5 (2.3)	25.5 (5.1)
Median of antigenic C1-INH, g/l (SD)	0.06 (0.17)	0.06 (0.03)	0.44 (0.13)
Median of antigenic C4, g/l (SD)	0.08 (0.07)	0.08 (0.07)	0.055 (0.06)
Median of functional C1-INH, % (SD)	< 15.6 (8.22)	< 15.6 (8.68)	< 15.6 (7.57)

Data about mean age of patients, symptom’s onset, diagnosis and diagnostic delay (expressed in years), and laboratory assessments. Antigenic C1-INH normal range: 0.21–0.39 g/l, antigenic C4 normal range: 0.1–0.4 g/l 1–11 years and 0.2–0.5 g/l > 11 years. Functional C1-INH normal range: 77.3–128.8%

Thirty-two patients accepted to fill up a questionnaire to collect data about attacks and triggers. A total of 368 attacks were reported with an attacks mean/patient of 10 angioedema attacks annually: the number of attacks ranged from 1 to 41 episodes per year. According to questionnaires, attacks of angioedema were most often subcutaneous swellings (46% attacks), followed by abdominal edema (32%) and larynx (22%). In 72.4% of patients, triggering factors preceded angioedema. Symptoms were precipitated by stress in 26.4% of patients, infections in 16.1%, physical triggers in 87.3%, 36.2% of patients mention dental treatment, 6.5%-menstruation and 43.0%-seasonal changes.

### Genetics

**Fig. 2:**
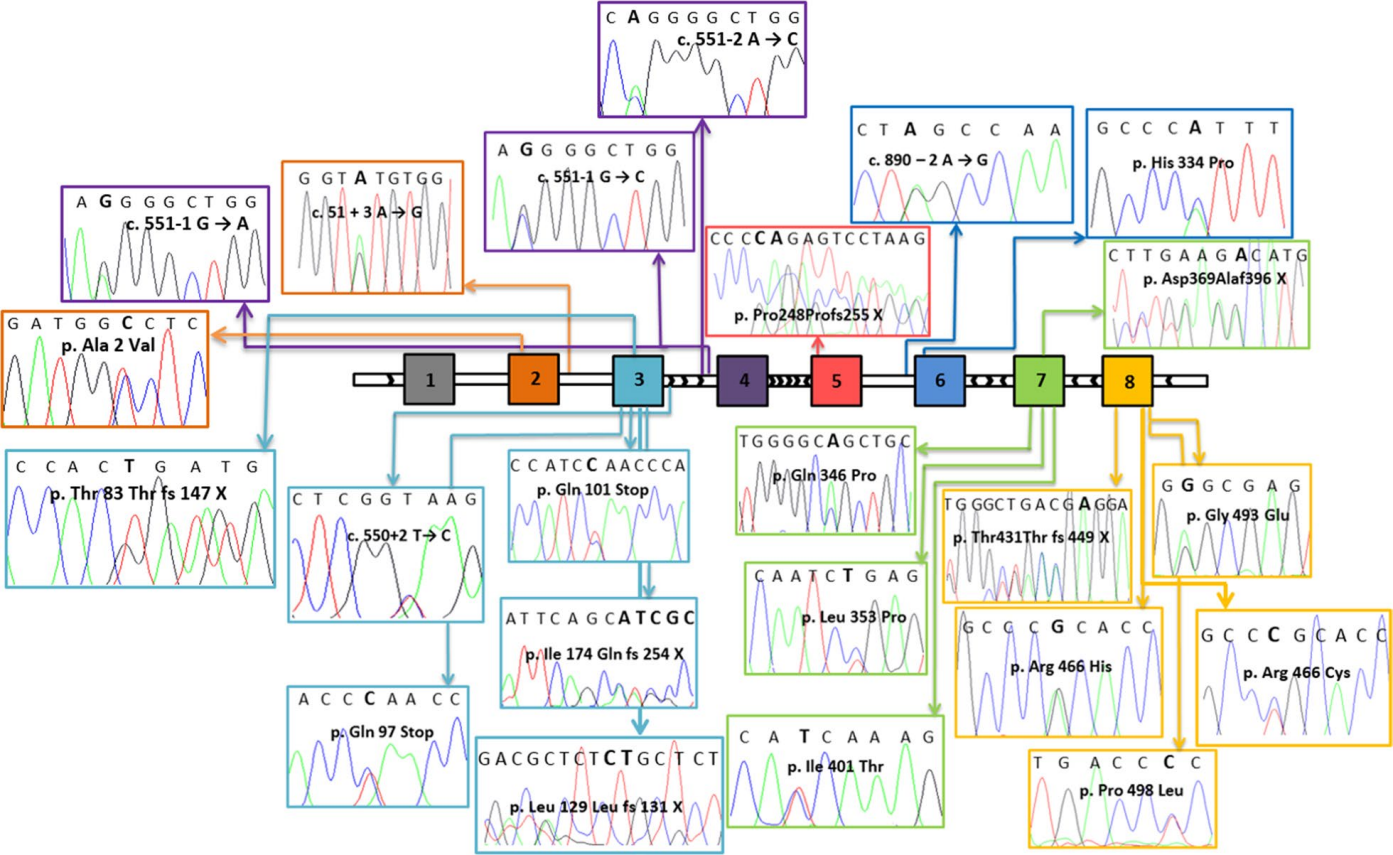
Mapping of *SERPING1 *variants.* SERPING1* variants found in Belarus patients are positioned within the gene map (exons are designated with numbered squares) by the help of arrowed lines and colours. The chromatogram relative to each variant is shown, indicating that all of them are present in heterozygosis. Variants localized in exons are indicated with the corresponding change in the translated amino acid residue, while variants localized in introns with the change on cDNA sequence. To process the data the Sequencing Analysis 5.2 software was used

All 64 patients underwent a molecular genetic analysis of the *SERPING1* gene. We found genetic variants associated with HAE in all DNA samples. Molecular genetic analysis identified 24 different variants: 9 missense (37.5%), 2 nonsense (8.3%), 1 large deletion (4.2%), 6 frameshift (25%) and 6 splicing defects (25%) (Fig. 2). Seven variants were not reported previously (Table 2) [3]: 4 frameshift variants, 2 in exon 3 (c.387_388delCT and c.520_524delATCGC), 1 in exon 5 (c.744_745delCA) and 1 in exon 8 (c.1293delA), and 3 missense variants, 1 in exon 6 (c.1001A > C) and 2 in exon 7 (c.1037A > C and c.1202T > C). Variants are described in relation to Reference Sequence NM_000062.2. All subjects were heterozygous for the mutations identified. De novo variants were found in 11 patients (17.2%).

**Table 2:** The spectrum of SERPING1 variants in Belarus patients

Number of affected patients	cDNA change (NM_000062.2)	Predicted effect on protein	ENST00000278407.8	Exon	Variant type	PolyPhen2°
1	c.5C > T	p.Ala2Val	rs185342631	2	Missense	0.987
2	c.51 + 3A > G		CS053487	2	Splicing	
3	c.249delT	p.Asp84Metfs*64		3	Frameshift	
1	c.289C > T	p.Gln97S*	CM128686	3	Nonsense	
1	c.301C > T	p.Gln101*		3	Nonsense	
1	c. 387_388delCT	p. Leu129Leufs131*2		3	Frameshift	
1	c.520_524del ATCGC	p.Ile174Glnfs254*81		3	Frameshift	
7	c.550 + 2 T > C		rs112666115	3	Splicing	
2	c.551-2A > C		rs113574262	4	Splicing	
2	c.551-1G > A			4	Splicing	
6	c.551-1G > C			4	Splicing	
3	del exon 4			4	Large del	
2	c.744_745delCA	p.Arg249Serfs*7		5	Frameshift	
2	c.890-2A > G		D0077: g.9903 A → G	6	Splicing	
1	c.1001A > C	p.His334Pro		6	Missense	0.969
1	c.1037A > C	p.Gln346Pro		7	Missense	0.996
4	c.1058 T > C	p.Leu353Pro		7	Missense	1
2	c.1106delA	p.Asp369Alafs*28	CD033556	7	Frameshift	
1	c. 1202 T > C	p.Ile401Thr		7	Missense	0.949
5	c.1293delA	p.Glu432Argfs*18		8	Frameshift	
7	c.1396C > T^a^	p.Arg466Cys	rs28940870	8	Missense	0.921
3	c.1397G > A^a^	p.Arg466His	rs121907948	8	Missense	0.66
5	c.1478G > A	p.Gly493Glu	CM022845	8	Missense	1
1	c.1493C > T	p.Pro498Leu		8	Missense	1

Gray filling specifies variants that have not been previously reported. Variants are described according to the HGVS-nomenclature (https://varnomen.hgvs.org). ^a^variant associated with C1-INH-HAE type II

### Access to prophylactic and on-demand therapy

Eight out of 64 patients were diagnosed before their first attacks, so they were not asked to fill out a questionnaire about LTP and ODT. Thirty-eight patients (67.8%) answered the questionnaire about therapy. Eleven patients answered that they did not use any treatment to resolve HAE attacks, they are waiting 2–4 days until the swelling will abates.

Thus, data about ODT used for the treatment of HAE attacks were collected in 27 patients. Only 9 patients were treated with appropriate drugs (2 with Icatibant or C1-INH concentrate and 7 with fresh frozen plasma infusion); 18 patients were treated with ineffective drugs for the disease: antihistamine (2 patients), steroid (5 patients), tranexamic acid (11 patients). Only 9 patients used Danazol as prophylactic therapy, 1 used tranexamic acid (Table 3).

**Table 3:** Questionnaire responses

Questionnaires number (% of total patients)	38 (67.9)
Prophylactic therapy n	11
Danazol (n)	9
Tranexamic (n)	1
Antihistamine (n)	1
Number of attack/year	368
Mean	10
Median	5.5
Min	1
Max	41
On-demand therapy ineffective n	18
Antihistamine (n)	2
Steroid (n)	5
Tranexamic acid (n)	11
On-demand therapy effective n	9
Icatibant or C1-INH concentrate (n)	2
Fresh frozen plasma infusion (n)	7

38 patients in the study completed a questionnaire about LTP and ODT. 32 patients filled up a questionnaire about attacks and triggers

## Discussion

Looking at the map illustrating the geographical distribution of C1-INH-HAE patients in Belarus, it is evident as most of them live in Minsk, where the Research Centre of Borovlyani is located. This indicates that patients living in or near Minsk can more easily reach the centre in order to undergo the necessary examinations and/or to have easier access to care. Also, Minsk and neighbouring areas have in their disposition a greater pathology knowledge base, access to relevant trainings and better developed health services. At the same time, in the Grodno region Belarussian National Public Organization "HAE Patients Care" is located, whose members actively help patients with provisional HAE come to Minsk for the diagnosis, with reflected in the number of patients identified in the Grodno region. The quantity is identical to the number of patients identified in the Minsk region. The remaining parts of Belarus report the lack of knowledge about HAE as well as ability to timely diagnose. This situation results in lower prevalence and increased diagnostic delay compared to data from surveys conducted in other countries [10–22].

Despite this situation, a high number of patients have been diagnosed in the last three years. The collaboration with Italian HAE centre of Milan, marked a significant improvement in the management of HAE.

Genetic data indicate that, although numerous and various *SERPING1* variants have been already reported in C1-INH-HAE, given the high number of novel variants identified in our study, the spectrum of variants responsible for this rare pathology is not completely characterized yet. Moreover, splicing defects amounted to 25%, which differs from the world data (about 10%) [29].

Data extrapolated from the answers to the questionnaire regarding therapeutic possibilities, suggest a serious deficiency in Belarus in accessing preventive therapy with Danazol: only 9 patients, indeed, declared to carry on a prophylactic treatment with such drug.

Evaluating the number of attacks reported by patients in the questionnaire, at least 11 out of 32 patients without prophylaxis had a number of attacks greater than 2 per month, above which the attenuated androgen therapy is indicated [23]. Furthermore, no patient reported short-term prophylactic therapy with attenuated androgens before surgical intervention, particularly in the oral cavity.

Regarding ODT for acute attacks, it is important to consider how a third of the patients (11 out of 38) did not answer the question about drugs used during acute attacks. This could be a result of inadequate patient training about the effective drugs for the treatment of HAE. Analysing 27 answers of the patients, it becomes obvious the poor adherence to guidelines for therapeutic management of acute attacks in Belarus. In fact, as many as 18 patients were treated with ineffective drugs for HAE: antihistamine, steroid, tranexamic acid. Only 9 patients were treated with appropriate drugs, 2 with icatibant or i.v. C1-INH concentrate and 7 with fresh frozen plasma infusion.

Icatibant or i.v. C1-INH concentrate are not available even at the national hospitals and not refunded by Belarusian Health System. Hence, the patients have to import the drugs from abroad on their own expense. Therefore, danazol, tranexamic acid and fresh frozen plasma infusion are used as the primary maintenance treatment in Belarus.

Our data show a lack of knowledge of on-demand HAE effective drugs both by patients and by health personnel. Lack of proper knowledge and education on patients' pathology lead to a condition of extreme vulnerability of the patients even in protected settings such as hospitals. Indeed, during the hereditary history taking, patients with HAE reported 12 deaths of their blood relatives due to laryngeal edema. Moreover, unfortunately, one patient, out of 64 presented in this study, died of angioedema in the hospital, because the provided therapy was not effective due to the lack of necessary medications.

## Conclusions

Belarus is an independent State formed in 1991. This is the first report of a HAE patients from Belarus, here well characterized from a genetic point of view. We found 24 different *SERPING1* variants, 7 have not been previously described. The lack of access to effective drugs (mainly for ODT) raises a major concern about the risks to have life-threatening laryngeal attacks, known to be the main disease specific cause of death in HAE patients [2]. The young Belarusian Health System have now other priorities such as control of infectious diseases and improvement of cancer and cardiovascular disease, before approaching rare diseases like HAE. Despite that, HAE management has improved, as seen from the high number of new diagnosis in the last 3 years. Next steps will be reducing the diagnostics delay, broader use of prophylactic and on-demand therapy as indicated in HAE guidelines [10].

## Data Availability

The authors confirm that all data underlying the findings are fully available without restriction. All relevant data are within the paper.
